# Brief online implicit bias education increases bias awareness among clinical teaching faculty

**DOI:** 10.1080/10872981.2021.2025307

**Published:** 2022-01-17

**Authors:** Janice Sabin, Grace Guenther, India J. Ornelas, Davis G. Patterson, C. Holly A. Andrilla, Leo Morales, Kritee Gurjal, Bianca K. Frogner

**Affiliations:** a Department of Biomedical Informatics and Medical Education, University of Washington, the University of Washington Center for Health Workforce Studies, UW School of Medicine, Seattle, WA, USA; b Research, University of Washington Center for Health Workforce Studies, Seattle, WA, USA; c Department of Health Services, School of Public Health, University of Washington, Seattle, WA, USA; d Department of Family Medicine, University of Washington, Seattle, WA, USA; e Research, University of Washington Center for Health Workforce Studies, WWAMI Rural Health Research Center, Seattle, WA, USA; f Department of Medicine, School of Medicine, Center of Health at the University of Washington, Seattle, WA, USA; g U.S. Department of Veterans Affairs, The Health Economics Resource Center (HERC), Menlo Park, CA, USA; h Department of Family Medicine, University of Washington (UW), UW Center for Health Workforce Studies, Seattle, WA, USA

**Keywords:** Implicit bias, continuing education, bias awareness

## Abstract

**Problem and Purpose:**

Healthcare provider implicit bias influences the learning environment and patient care. Bias awareness is one of the key elements to be included in implicit bias education. Research on education enhancing bias awareness is limited. Bias awareness can motivate behavior change. The objective was to evaluate whether exposure to a brief online course, *Implicit Bias in the Clinical and Learning Environment*, increased bias awareness.

**Materials and Methods:**

The course included the history of racism in medicine, social determinants of health, implicit bias in healthcare, and strategies to reduce the impact of implicit bias in clinical care and teaching. A sample of U.S. academic family, internal, and emergency medicine providers were recruited into the study from August to December 2019. Measures of provider implicit and explicit bias, personal and practice characteristics, and pre–post-bias awareness measures were collected.

**Results:**

Of 111 participants, 78 (70%) were female, 81 (73%) were White, and 63 (57%) were MDs. Providers held moderate implicit pro-White bias on the Race IAT (Cohen’s *d* = 0.68) and strong implicit stereotypes associating males rather than females with ‘career’ on the Gender-Career IAT (Cohen’s *d* = 1.15). Overall, providers held no explicit race bias (Cohen’s *d* = 0.05). Providers reported moderate explicit male-career (Cohen’s *d* = 0.68) and strong female-family stereotype (Cohen’s *d* = 0.83). A statistically significant increase in bias awareness was found after exposure to the course (*p* = 0.03). Provider implicit and explicit biases and personal and practice characteristics were not associated with an increase in bias awareness.

**Conclusions:**

Implicit bias education is effective to increase providers’ bias awareness regardless of strength of their implicit and explicit biases and personal and practice characteristics. Increasing bias awareness is one step of many toward creating a positive learning environment and a system of more equitable healthcare.

## Introduction

The 2003 Institute of Medicine landmark report, *Unequal Treatment: Confronting Racial and Ethnic Disparities In Healthcare*, was the first report to compile indisputable evidence of healthcare disparities and suggest implicit bias contributes to racial and ethnic healthcare disparities [1]. Implicit biases are positive or negative attitudes and stereotypes that are not available to introspection but influence thoughts, decisions, and actions toward others [2]. Explicit biases are attitudes and beliefs that make up our belief system and that we can report [2]. The first studies to investigate implicit bias in healthcare focused on identifying whether implicit bias exists among healthcare providers and if bias impacts patient care [3–5]. Since then, studies have found that provider implicit racial bias is associated with less patient-centered communication, less patient trust of their clinician [6], poorer quality of clinical interactions, and disparities in pain management and other areas [6–10]. Research on implicit bias in healthcare has expanded to include gender [11], sexuality [12], weight [13], mental health [14], socio-economic status [15], and many other areas.

Gender bias in medicine persists [16], and impacts patient care, trainee assessment, and organizational climate. Liaudat (2018) found that despite the equal prevalence of chest pain in ambulatory care, men were 2.5 times more likely to be referred to a cardiologist than women [17]. In a review of gender bias in resident physician assessment, five of nine studies found that gender bias potentially influenced assessment of medical residents [18]. One study found that gender bias leads to negative experiences for female surgical trainees and that female trainees in male-dominant specialties ‘may leave medicine/retire early’ [19].

There has been a proliferation of implicit bias education for healthcare providers [20–22]. Brief education on implicit bias has a positive effect on increasing diversity in medical residency admissions and reducing bias toward women in medicine [11,23]. Implicit bias curricula can be a tool to promote social justice [24]. Not enough is known about whether healthcare providers’ implicit and explicit biases and personal and practice characteristics affect uptake and application of implicit bias education. Sukhera & Watling [21] created a Framework for Integrating Implicit Bias Education into the Health Professions that outlines the importance of increasing bias awareness, creating a safe non-threatening learning context, increasing knowledge of the science of implicit bias, showing how implicit bias influences provider behaviors and patient outcomes, improving efforts to overcome bias, and enhancing awareness of how bias influences others [21]. Evaluation on whether these interventions increase healthcare providers’ bias awareness is limited.

Becoming aware of bias and accepting that bias exists is an initial step towards reducing personal discrimination and promoting culture change [25,26]. Awareness of bias can lead to an individual being more receptive to feedback and recognizing acts of subtle bias as acts of racial discrimination [26]. Awareness of bias may reduce racial discrimination in healthcare settings, which has been shown to increase medical student observers’ bias [27]. We created innovative education on implicit bias designed for clinicians who teach that included many of the elements of the Framework [21] and measured pre- and post-bias awareness.

The course content was purposefully developed to serve as foundational information for academic health care providers who teach. The 40-min course, *Implicit Bias in the Clinical and Learning Environment*, included content on the history of racism in medicine, social determinants of health (SDOH), evidence of health and healthcare disparities, the science of implicit bias, how bias operates in the clinical and learning environments, and strategies to mitigate the impact of implicit bias. Online delivery of the course provided a safe learning environment and extended access to learning [21]. Our research questions were as follows: (1) Following completion of the course, does provider bias awareness change? And (2) Are provider implicit and explicit race and gender biases and personal and practice characteristics associated with bias awareness change? Our evaluation focused on Kirkpatrick Level 2 learning impact [28] using a pre–post-bias awareness measure [11] to assess providers' uptake of course content and determine whether providers’ exposure to course content changed bias awareness (an attitude). We hypothesized that the course would improve bias awareness and that implicit race and gender bias might impede an increase in bias awareness.

## Materials and methods

### Sample and recruitment

From August to December 2019, we used a cold email blast method to recruit a sample of medical doctors (MDs), nurse practitioners (NPs), and physician assistants (PAs) who were clinical teaching faculty in U.S. academic medical institutions using public information from institutional websites. Using the *Association of American Medical Colleges (AAMC) U.S. Medical School Faculty by Medical School and Department Type, 2018* [29], we chose four medical schools, two public and two private, two nursing schools within each of the nine U.S. Census Divisions, and 19 U.S. PA programs, purposefully striving for geographic diversity. We identified publicly available emails on university/program websites by departments based on their roles in three specialties providing primary care (internal medicine, family medicine, and gerontology) as well as emergency medicine. Because NP and PA subspecialty could not be determined, we invited all clinical faculty from these schools to participate. Our prospective participant email list included 7,671 emails recruited from a total of 144 schools of medicine, nursing, and physician assistant, representing all nine U.S. Census Sub-Regions. Included in our recruitment list were 90 medical schools (including large, small, public, and private), 35 nursing schools, and 19 physician assistant programs. We recruited an average 16 schools from each of nine U.S. Census Sub-Regions, for about 10 medical schools, 4 nursing schools, and 2 physician assistant programs representing each subregion.

Eligibility criteria included both direct patient contact and teaching or mentoring students/trainees. Participants were offered a $40.00 gift card and certificate of course completion as incentive. Our institution’s Human Subjects Institutional Review Board approved the study.

### *Implicit bias education*: implicit bias in the clinical and learning environment

The need for brief online implicit bias education for teaching faculty was driven by medical student identification of deficits in faculty proficiency in this area. The course was developed by faculty with expertise in adult learning, implicit bias in healthcare, online education, academic clinicians who teach or mentor, regional affiliate faculty, school of medicine leadership, and others. In developing the course, we aimed to be brief, which busy clinicians identified as necessary due to high workloads. We wanted to expose faculty to many concepts, define common terminology, and create foundational knowledge among all providers in a large and geographically diverse system. We believed the combination of the history of racism in medicine, evidence of social determinants of health, and the science of implicit bias would form a powerful and necessary knowledge foundation for academic clinicians who teach. We presented common terms and definitions, evidence that implicit bias is common, and the way our minds work [30]. We presented evidence that implicit bias is contagious [31], which underscores that the point that clinicians who teach are important role models. We introduced the concepts of split-second judgements [32], in-group favoritism [30], and stereotype threat [33] and showed how they impact clinical care and teaching. We provided strategies to mitigate bias such as removing discretion from decision-making and collecting data to identify inequities. The course addressed three learning objectives: (1) Define implicit bias and how it is manifested in healthcare; (2) Recognize how implicit bias may be operating in the clinical setting and learning environment; and (3) Apply strategies that can be used to minimize the impact of implicit bias on teaching and clinical practice. Our evaluation used a pre–post-bias awareness measure [11] to determine whether providers’ bias awareness was impacted. The course is publicly available for all to use (https://catalyst.uw.edu/webq/survey/dolson/394766).

### Three-part one-hour survey and education intervention

**Part 1**. Participants completed an online survey that asked about personal characteristics, an 8-item bias awareness measure [11], the Race Implicit Association Test (IAT) and Gender-Career IAT [34], and corresponding questions about explicit race and gender bias (described below).

**Part 2**. Participants took the online course.

**Part 3**. Participants completed a second survey that included questions about practice characteristics (described below) and the same 8-item bias awareness measure [11].

### Measures

#### Demographic and practice characteristics

Demographic information included age, sex assigned at birth, gender identity, ethnicity, and race. Practice characteristics included area of practice, degree, years in practice, years in position, number of patients per week, direct patient contact hours, continuing education (CE) on racial and ethnically diverse populations, and gender equity.

#### Bias awareness

We used a bias awareness measure developed by Girod et al. [11] to assess pre–post-bias awareness change among academic physicians following an implicit bias educational intervention on gender bias in faculty advancement for hiring committees. The authors concluded that an immediate change in awareness of bias demonstrated that participants had ‘absorbed’ the content of their educational intervention. The items focused on three content domains: personal, societal, and in-healthcare using a 6-point scale ranging from ‘1 = Strongly Agree’ to ‘6 = Strongly Disagree.’ The eight statements were as follows: *1. In most situations, I am objective in my decision-making, 2. Biases do not usually influence my decision-making, 3. Men and women vary in the types of biases they have against other people, 4. People in today’s society tend to treat people of different social groups [i.e., race, gender, and class] equally, 5. Society has reached a point where all people, regardless of background, have equal opportunities for achievement, 6. In healthcare, bias against others is no longer a problem in the area of patient care, 7. In healthcare, bias against others is no longer a problem in the area of training, and 8. In healthcare, bias against others is no longer a problem in the area of a diverse workforce*. The higher the score, the more awareness an individual demonstrated. The 8-item bias awareness measure has good internal consistency, with a Cronbach alpha coefficient of .78. The mean inter- item correlation is .31.

#### Implicit measures

The IAT is a widely used computer-based test that measures the relative strength of positive and negative associations toward one social group compared with another. The Race IAT asks test takers to sort and pair facial images of the target concept and value-laden words that represent the concepts of good or bad to assess automatic associations of these concepts. The difference in the time it takes to quickly sort and pair the images and words correctly shows the strength of automatic association. The Gender-Career IAT measures the association between gender (male versus female) and the concepts of career versus family. The IAT was used as a metric and individual IAT score feedback was not provided. At the end of the survey, participants were given the link to Project Implicit (https://implicit.harvard.edu) to learn more about implicit bias and take IATs, if they chose. We calculated the IAT effect according to the 2003 published scoring algorithm [35].

#### Explicit measures

We used explicit measures that corresponded to the IAT measures. The explicit measure for the Race IAT was as follows: Which statement best describes you? *1. I strongly prefer Black People compared to White People; 2. I moderately prefer Black People compared to White People; 3. I slightly prefer Black People compared to White People; 4. I like Black People and White People equally; 5. I slightly prefer White People compared to Black People; 6. I moderately prefer White People compared to Black People; and 7. I strongly prefer White People compared to Black People*. For the Gender IAT, we used two separate explicit measures: one that asked about association of gender (male versus female) with career and one that asked about association of gender (male versus female) with family.

### Analysis

We used descriptive statistics (means, standard deviations, and frequencies) to characterize the sample. We used ANOVA, Pearson correlation, and *t*-tests to determine associations and statistically significant differences in bias awareness change across participants and by participant implicit and explicit bias scores and demographic and practice characteristics. To determine the effect size of implicit and explicit measures, we used Cohen’s *d*, interpreted as *d* = 0.2 is considered a ‘small’ effect size, *d* = 0.5 a ‘medium’ effect size, and *d = *0.8 a ‘large’ effect size [36]. We conducted statistical analyses using SAS (version 9.4; Cary, NC: SAS) and IBM SPSS Statistics for Macintosh (version 26.0; Armonk, NY: IBM Corp).

## Results

Our sample of *N* = 111 included provider representation from all nine U.S. Census Sub-Regions. The characteristics of the sample (*N* = 111) are presented in Table 1. Of 111 participants, 78 (70%) were female, 81 (73%) were White, and 63 (57%) were MDs. We found that 74 (67%) participants had engaged in CE on working with diverse populations and 50 (45%) had engaged in CE on gender equity in the past year. Providers held moderate levels of implicit pro-White bias (Cohen’s *d* = 0.68) (Table 2). There was a significant difference in strength of implicit race bias based on participant race (*p* < 0.001). White and Asian participants held strong implicit bias favoring White Americans versus Black/African Americans (Cohen’s *d* = 0.93 and Cohen’s *d* = 1.93, respectively). Black/African American participants, 83% (*N* = 10) of whom lived in the South, held a strong implicit bias favoring African Americans over White Americans (Cohen’s *d* = 0.86). Overall, by region, Race IAT scores favored White Americans over African Americans, but there were differences in magnitude of this implicit race bias by region (*p* = 0.04). Participants in the Northeast (Cohen’s *d* = 1.05) and Midwest (Cohen’s *d* = 1.19) held strong implicit race bias; in the West, moderate implicit race bias (Cohen’s *d* = 0.70); and in the South, weak implicit race bias (Cohen’s *d* = 0.35). We chose not to apply a Bonferroni correction to these tests due to concerns that it has been shown to be overly conservative. Other possible adjustments, such as using a *p*-value that yields an expected number of false positive less than 1, do not change the significance of the findings reported except for the Census Division association (*p* = 0.04) in Table 2. The direction of this finding makes intuitive sense and suggests an area for further research on regional variations in providers' attitudes about race. We found strong implicit stereotypes associating males versus females with career on the Gender-Career IAT (Cohen’s *d* = 1.15). This was consistent across participant gender, race, and geographic region.

**Table 1.** Healthcare provider characteristics (*N* = 111)

Variables	*N* (%)
**Age**	
30–39	38 (34.2)
40–49	29 (26.1)
50–59	25 (22.5)
60+	19 (17.1)
**Gender identity**	
Male	33 (29.7)
Female	78 (70.3)
**Ethnicity**	
Hispanic or Latino	8 (7.2)
Non-Hispanic or Latino	103 (92.8)
**Race **	
White	81 (73.0)
Asian	10 (9.0)
Black	12 (10.8)
Mixed/other	8 (7.2)
**Provider type**	
Medical doctor (MD)	63 (56.8)
Nurse practitioner (NP)/physician assistant (PA)	23 (20.7)
Other (registered nurse, clinical nurse educator, other)	25 (22.5)
**U.S. Census Region**	
Northeast	25 (22.5)
Midwest	22 (19.8)
South	45 (40.5)
West	19 (17.1)
**Healthcare system**	
Academic Healthcare System	80 (72.7)
Community Healthcare System	21 (19.1)
Other	9 (8.1)
**Continuing education past year: race and diversity**	
Yes	74 (66.7)
No	37 (33.3)
**Continuing education past year: gender equity**	
Yes	50 (45.0)
No	61 (55.0)
	**Mean (SD)** **or %**
**Years in practice**	18.6 (12.02)
**Years current position **	8.4 (7.64)
**# Patients/week direct clinical contact **	37.5 (30.3)

**Table 2.** Implicit race and gender attitudes overall and by sample characteristics (*N* = 111)

		Implicit race^a^			Implicit gender^a^		
	*N*	M (SD)	Cohen’s *d*^b^	*p*^c^	M (SD)	Cohen’s *d*	*p*
**Total sample**		0.30 (0.43)	0.68	**<0.001**	0.38 (0.33)	1.15	**<0.001**
**Age**				0.77			0.40
30–39	38	0.25 (0.42)	0.60		0.40 (0.27)	1.52	
40–49	29	0.36 (0.43)	0.84		0.30 (0.35)	0.86	
50–59	25	0.28 (0.50)	0.70		0.45 (0.38)	1.18	
60+	19	0.34 (0.4)	0.85		0.39 (0.36)	1.08	
**Self-reported gender identity**				0.74			0.41
Male	33	0.41 (0.44)	0.93		0.41 (0.30)	1.37	
Female	73	0.26 (0.42)	0.62		0.37 (0.35)	1.12	
**Ethnicity**				0.80			0.46
Hispanic or Latino	8	0.27 (0.45)	0.60		0.48 (0.39)	1.23	
Non-Hispanic or Latino	103	0.30 (0.44)	0.68		0.37 (0.33)	1.12	
**Race **				**<0.001**			0.80
White	81	0.37 (0.40)	0.93		0.36 (0.35)	1.11	
Asian	10	0.54 (0.28)	1.93		0.43 (0.28)	1.54	
Black	12	−0.25 (0.29)	0.86		0.42 (0.30)	1.40	
Mixed/other	8	0.15 (0.42)	0.34		0.45 (0.35)	1.29	
**Provider type**				0.71			0.39
Medical doctor (MD)	63	0.32 (0.43)	0.74		0.36 (0.33)	1.09	
Nurse practitioner (NP)/physician assistant (PA)	23	0.35 (0.36)	0.97		0.36 (0.33)	1.00	
Other	25	0.24 (0.50)	0.48		0.46 (0.32)	1.44	
**U.S. Census Region**				**0.04**			0.73
Northeast	25	0.41 (0.39)	1.05		0.41 (0.34)	1.21	
Midwest	22	0.43 (0.36)	1.19		0.41 (0.28)	1.46	
South	45	0.17 (0.45)	0.38		0.38 (0.36)	1.06	
West	19	0.33 (0.47)	0.70		0.33 (0.47)	1.21	
**Healthcare system**				0.84			0.33
Academic Healthcare System	80	0.31 (0.40)	0.78	0.21	0.39 (0.32)	1.22	
Community Healthcare System	21	0.32 (0.58)	0.55		0.39 (0.37)	1.05	
Other	8	0.23 (0.40)	0.56		0.22 (0.41)	0.55	
**Continuing education past year: race**				0.22			0.16
Yes	74	0.27 (0.46)	0.59		0.35 (0.35)	1.00	
No	37	0.36 (0.38)	0.95		0.43 (0.31)	1.39	
**Continuing education past year**: **gender equity**				0.43			0.30
Yes	50	0.29 (0.40)	0.73		0.40 (0.36)	1.39	
No	61	0.31 (0.46)	0.67		0.35 (0.35)	1.00	

^a^For implicit measures, a positive score favors White on Race IAT and male and career on the Gender IAT, and a negative score indicates favoring Black/African American and female with family.

^b^Cohen *d* values are interpreted as follows: *d* = 0.2, small effect; *d* = 0.5, medium effect; and *d* = 0.80, large effect, Cohen, 1988.

^c^ANOVA and *t*-test of significance [value = 0]. Boldface indicates statistical significance (p<0.05).

There were statistically significant differences in explicit race bias by participant race (Table 3). Black participants moderately favored Black/African Americans (Cohen’s *d* = 0.54), while other groups (White, Mixed/Other) held weak pro-White explicit race bias and Asian participants repored no explicit race bias. There was a significant difference in explicit race bias among those who had continuing education on working with diverse populations, but this difference was not meaningful because their means hovered at zero (0.01 and 0.08, respectively). On two separate explicit gender bias questions (gender-career and gender-family), participants reported a moderate to strong explicit stereotype associating males rather than females with the concept of career (Cohen’s *d* = 0.68) and females with the concept of family (Cohen’s *d* = 0.83). There were no significant differences in explicit gender biases, except among those who worked in community healthcare systems who only very slightly associated females rather than males with family (Cohen’s *d* = 0.25).

**Table 3.** Explicit race and gender attitudes overall and by sample characteristics (*N* = 111)

		Explicit Race^a^			Explicit career^b,c^				Explicit family^b, c^		
	*N*	M (SD)	Cohen’s *d*	*p*	M (SD)	Cohen’s *d*	*p*	M (SD)		Cohen’s *d*	*p*
**Total sample**	111	0.04 (0.74)	0.05	0.61	0.66 (0.97)	0.68	<0.001	0.83(1.00)		0.83	<0.001
**Age**				0.23			0.95				0.77
30–39	38	−0.16 (0.89)	0.18		0.73 (1.04)	0.96		−0.79 (1.12)		0.71	
40–49	29	0.10 (0.67)	0.15		0.62 (1.05)	0.92		−0.97 (0.82)		1.18	
50–59	25	0.12 (0.67)	0.30		0.67 (0.82)	0.81		−0.88 1.03)		0.97	
60+	19	0.22 (0.55)	0.41		0.58 (0.96)	0.6		−0.67 (0.97)		0.69	
**Self-reported gender identity**				0.18			0.86				0.08
Male	32	0.16 (0.63)	0.25		0.5 (0.95)	0.53		−0.82 (0.81)		1.01	
Female	78	−0.01 (0.78)	0.01		0.73 (0.97)	0.74		−0.84 (1.07)		0.79	
**Ethnicity**				0.28			0.11				0.66
Hispanic or Latino	8	0.0 0(0.93)	0		−0.88 (1.36)	0.74		−1.38 (1.06)		1.30	
Non-Hispanic or Latino	103	−0.04 (0.73)	0.04		0.64 (0.94)	0.68		−0.79 (0.98)		0.81	
**Race **				**0.01**			0.40				0.58
White	81	0.17 (0.54)	0.31		0.59 (0.90)	0.66		−0.86 (0.9)		0.96	
Asian	9	0.0 (0.70)	0		1.10 (1.20)	0.83		−1.10 (0.74)		1.49	
Black	12	−0.67 (1.23)	0.54		0.83 (1.34)	0.62		−0.67 (1.72)		0.39	
Mixed/other	8	−0.25 (1.04)	0.24		0.50 (0.76)	0.66		−0.50 (0.76)		0.66	
**Provider type**				0.77			0.36				0.13
Medical doctor (MD)	63	0 .00 (0.76)	0		0.61 (0.93)	0.66		−1.00 (0.91)		1.1	
Nurse practitioner (NP)/physician assistant (PA)	23	0.13 (0.87)	0.15		0.91 (1.08)	0.50		−0.56 (1.12)		0.50	
Other	25	0.04 (0.54)	0.07		0.54 (0.98)	0.55		−0.67 (0.96)		0.70	
**U.S. Census Region**				0.23			0.55				0.97
Northeast	25	0.13 (0.54)	0.33		0.72 (0.89)	0.81		−0.76 (0.93)		0.82	
Midwest	22	0.23 (0.69)	0.24		0.55 (0.67)	0.82		−0.81 (0.87)		0.93	
South	45	−0.13 (0.89)	0.15		0.78 (1.22)	0.64		−0.86 (1.19)		0.72	
West	19	0.11 (0.57)	0.19		0.42 (0.61)	0.69		−0.89 (0.74)		0.74	
**Healthcare system**				0.94			0.54				**0.01**
Academic Healthcare System	80	0.03 (0.75)	0.04		0.65 (1.00)	0.65		−1.00 (0.93)		1.06	
Community Healthcare System	21	0.05 (0.89)	0.05		0.48 (0.75)	0.64		−0.28 (1.15)		0.25	
Other	9	0.11 (0.33)	0.11		0.89 (0.93)	0.98		−0.75 (0.71)		1.06	
**Continuing education past year: diverse populations**				**0.01**							0.52
Yes	74	0.01 (0.82)	0.01		0.53 (0.90)	0.59		−0.75 (1.02)		0.74	
No	37	0.08 (0.55)	0.15		0.92 (1.06)	0.86		−1.00 (0.93)		1.08	
**Continuing education past year: gender equity**				0.33			0.86				0.40
Yes	50	0.14 (0.79)	0.17		0.56 (0.99)	1.38		−0.92 (0.93)		0.99	
No	61	0.05 (0.69)	0.07		0.74 (0.94)	1		−0.77 (1.05)		0.96	

^a^For the three explicit measures, a positive score favors White on the race measure. For the two gender explicit measures, a positive score associates male with career, and a negative score associates female with family.

^b^ANOVA and *t*-test of significance [value = 0]. Boldface indicates statistical significance (p<0.05).

^c^Cohen *d* values are interpreted as follows: *d* = 0.2, small effect; *d* = 0.5, medium effect; and *d* = 0.80, large effect, Cohen, 1988.

We found a significant increase in bias awareness after taking the course (*p* = 0.03) (Table 4). This change was driven by participants’ change in awareness of societal inequity (*p* < 0.00), indicating an increased awareness that society has not ‘reached a point where all people, regardless of background, have equal opportunities for achievement.’ There were no significant differences in bias awareness change by participant and practice characteristics.

**Table 4.** Pre- and post-course bias awareness (*N* = 111)

		Pre	Post	
Bias awareness score^a^	*N*	M (SD)	M (SD)	*p*^b^
In most situations, I am objective in decision-making	111	2.08 (0.99)	2.09 (0.99)	0.88
Biases do not usually influence my decision-making	111	2.91 (1.29)	2.86 (1.3)	0.52
Men and women vary in the types of biases they have against people	109	2.56 (1.15)	2.75 (1.12)	0.08
People in today’s society tend to treat people of different social groups (i.e., race, gender, and class) equally	111	4.38 (1.56)	4.54 (1.6)	0.29
Society has reached a point where all people, regardless of background, have equal opportunities for achievement	111	5.04 (1.34)	5.26 (1.2)	**0.00**
In healthcare, bias is no longer a problem in patient care	111	5.41 (0.88)	5.32 (0.87)	0.18
In healthcare, bias is no longer a problem in training	110	5.08 (1.17)	5.15 (1.05)	0.42
In healthcare, bias is no longer a problem in diverse workforce	114	5.14 (1.18)	5.14 (1.11)	0.89
**Bias awareness difference**	**N**	**M (SD)**	**M (SD)**	***p***
Composite (8-item average)	111	4.06 (0.76)	4.14 (0.73)	**0.03**
**Bias awareness difference by domain**				
Bias awareness personal	111	2.50 (1.01)	2.47 (1.04)	0.67
Bias awareness societal	111	4.00 (0.95)	4.48 (0.78)	**0.00**
Bias awareness in healthcare	111	5.19 (0.99)	5.22 (0.96)	0.86

^a^Bias awareness score: 1 = strongly agree, 2 = moderately agree, 3 = slightly agree, 4 = slightly disagree, 5 = moderately disagree, and 6 = strongly disagree.

^b^Pre- and post-bias awareness score difference, one-sample *t*-test, *t*-test value = 0. Boldface indicates statistical significance (p<0.05).

Implicit race attitudes were significant but weakly correlated with their corresponding explicit race attitude measure (*r* = 0.26, *p* = 0.01), suggesting that these are related but distinct attitudes (Table 5). Implicit measures of gender bias were not associated with explicit attitudes of gender-career (*r* = 0.18, *p* = 0.06) and gender-family (*r* = –0.13, *p* = 0.18) bias. Implicit and explicit biases were not associated with change in bias awareness.

**Table 5.** Implicit and explicit bias and bias awareness change (*N* = 111)

	Implicit race	Implicit gender	Explicit race	Explicit career	Explicit family	Bias awareness difference
**Implicit race**	1					
**Implicit gender**	.258**	1				
**Explicit race**	.207*	.055	1			
**Explicit career**	.024	.180	.082	1		
**Explicit family**	.002	−.130	−.196**	−.407**	1	
**Bias awareness difference**	.129	−.021	−.069	−.104	−.071	1

Pearson correlation: **Correlation is significant at 0.01 level, *correlation is significant at 0.05 level.

## Discussion

As we expected, exposure to the course, *Implicit Bias in the Clinical and Learning Environment*, resulted in increased bias awareness among academic healthcare providers. Our hypothesis that the strength of provider implicit bias would impact bias awareness change was not supported. An important contribution made by this study is that exposure to the course content increased providers’ bias awareness, even among this motivated and engaged sample of providers, regardless of provider race or gender biases and personal and practice characteristics. We conclude that the meaningful course content and brief online format can be useful to increase bias awareness among healthcare providers regardless of their biases or other characteristics. Increased awareness is a critical component of implicit bias education aimed at increasing healthcare equity [21]. Future research is needed with a nationally representative sample of academic providers who teach to determine whether implicit bias education can impact all providers’ awareness of bias.

On average, all participants, with the exception of Black/African Americans, held implicit race bias favoring Whites, similar to published literature on implicit bias among MDs and other clinicians [3,4,9,37]. One study found that African American MDs hold no implicit race bias [5], while our current study found implicit beliefs among Black providers that favor Black/African Americans. Participants from the South, which was the only region with representation of Black/African American providers, held weak implicit race bias, while participants from other regions held moderate to strong implicit bias favoring Whites.

Implicit gender bias can lead to inequities in patient care and underrepresentation of women in the workforce [7,9,38]. Similar to other studies [39], the majority of participants regardless of gender held strong implicit gender bias associating males rather than females with the concept of career. Less than half of participants had recent CE on gender equity. Given strong gender bias among providers and lack of female representation in leadership roles in academic medicine [11], gender bias in hiring [38,40] and gender equity continuing education is an area of immediate need. This education should aim for improving equity in the healthcare workforce, patient care, women represented in leadership, learning environments, and all aspects of daily life.

Increase in awareness of societal inequities may have been due to a powerful combination of content that devoted time to the history of racism in medicine, SDOH, evidence of the role of implicit bias in healthcare inequities, and strategies to mitigate the impact of bias in teaching and practice. It is important to note that the study was completed prior to the events of 2020, such as high-profile police killings of Black men and women, world-wide expansion of the Black Lives Matter movement, and the COVID-19 pandemic all of which heightened society’s awareness of social injustice and health inequity.

Awareness in the domain of personal bias did not change after taking the course. Participants believe that they are objective in their decision-making and that personal biases do not usually influence their decision-making both before and after the course. Providers believe they are objective, and they may be, but implicit bias by its very nature is imperceptible to the individual. Providers held little explicit race bias which may give a sense of immunity from all types of bias when estimating their own objectivity in decision-making.

Participants’ awareness of bias in-healthcare did not increase. It is notable that participants scored high on awareness of bias in society and in-healthcare at baseline. Two-thirds of our participants had recent CE on working with diverse populations and thus had familiarity with some of the course content. Healthcare providers who are less familiar with the course content may be more impacted by exposure to the course.

## Limitation

Our study has several limitations. First, our convenience sample is not a representative sample of academic providers who teach. Providers who chose to complete our 1-h research study on implicit bias in healthcare are a self-selected sample that is likely highly interested and engaged in the topic of health equity. Our findings among this self-selected sample may not generalize to all providers. Future research should assess the benefits and lasting effects of implicit bias education with a representative sample of providers. In our analysis, we did not disaggregate data by provider type because some of the categories would be too small to be meaningful. Future larger-scale studies should assess how implicit bias education impacts various disciplines and, if necessary, the curriculum should be tailored to the discipline.

There are similarities and differences between racial and gender characteristics of our sample compared to the U.S. primary care provider population (Supplemental Table 1). Compared to national data on primary care provider MDs and NPs, our MD and NP samples comprised more female and Hispanic people. In addition, our MD sample was more diverse than national samples, with less White MDs, less Asian MDs, and more Black MDs. Our NP sample was less White than national samples, had more Asian NPs, and had a similar number of Black NPs. Another limitation to this study is that brief online, one-time, implicit bias education may not have lasting effects [41,42]. One study found that one-time education can have a long-term impact on awareness of racial bias in medicine and that learners applied what they learned to advance equity [43]. Lasting effects of implicit bias education are a largely uncharted area in need of further research. In addition, further sub-analysis by participant race/ethnicity was prohibited due to small sample sizes.

## Implications

Brief implicit bias education that includes the science of implicit bias, the history of racism in medicine, and bias mitigation strategies can increase providers’ awareness of bias. Brief implicit bias education is one of the many types of equity education that can be useful to become more aware of bias, which is an essential component of implicit bias education [21,24] and a precursor of behavior change [44]. Implicit race and gender bias exists even among providers who are motivated to engage in the topic and have had previous training. These biases do not appear to be related to the benefits of the education, which suggests that regardless of the strength of implicit bias, providers’ awareness can increase. Among the many institutions represented in this study, the prevalence of gender equity education lags far behind the prevalence of diversity and inclusion education. Given the well-documented gender inequities in academic medicine, education on gender inequities across all levels of training and professional development is an area in need of immediate attention.

## Conclusion

Implicit bias among providers is associated with poor clinical interactions and disparities in healthcare [6,7,9,10] but does not appear to impact the benefits of implicit bias education. The AMA’s recent strategic plan for 2021–2023 includes developing and disseminating health equity and anti-racism curricula for faculty as part of a road map for action and accountability for racial justice and health equity [45]. Implicit bias education that increases healthcare providers’ bias awareness is one step of many steps toward creating a positive learning environment and a system of more equitable healthcare. Future research on implicit bias education should examine whether there are lasting effects of implicit bias education and whether provider bias awareness is associated with behavior change and ultimately improved health outcomes.

## Supplementary Material

Supplemental Material
