# Supplementary material for: Brief online implicit bias education increases bias awareness among clinical teaching faculty
Source: Med Educ Online. 2022 Jan 17;27(1):2025307. doi: 10.1080/10872981.2021.2025307 (PMC8765255; doi:10.1080/10872981.2021.2025307)
Supplement: Supplemental Material [file ZMEO_A_2025307_SM0970.docx]

|  | **Study Sample** | **National Sample** |
| --- | --- | --- |
|  | % | % |
| **Gender Identify** |  |  |
| Female MD | 55.6 | 45.0 |
| Female NP | 91.3 | 85.1 |
|  |  |  |
| **Ethnicity** |  |  |
| Hispanic MD | 9.8 | 5.9 |
| Hispanic NP | 5.3 | 4.5 |
|  |  |  |
| **Race MD** |  |  |
| White | 68.9 | 72.5 |
| Asian | 6.3 | 11.2 |
| Black | 12.6 | 6.8 |
|  |  |  |
| **Race NP** |  |  |
| White | 73.9 | 84.0 |
| Asian | 13.0 | 4.1 |
| Black | 5.3 | 5.7 |

**Supplemental Table 1. Race, Ethnicity and Gender of Our Sample Compared to National Sample**

**National sample sources:**

Xierali IM, Nivet MA. The Racial and Ethnic Composition and Distribution of Primary Care Physicians. *Journal of health care for the poor and underserved.* 2018;29(1):556-570.

Petterson S, McNellis R, Klink K, Meyers D, Bazemore AJ. The State of Primary Care in the United States: A Chartbook of Facts and Statistics. In. Washington DC: Robert Graham Center; January 2018.
